# Transcriptional and Post-Transcriptional Regulation of the *Escherichia coli luxS* mRNA; Involvement of the sRNA MicA

**DOI:** 10.1371/journal.pone.0013449

**Published:** 2010-10-18

**Authors:** Klas I. Udekwu

**Affiliations:** Department of Biology, Emory University, Atlanta, Georgia, United States of America; University of Hyderabad, India

## Abstract

**Background:**

The small RNA (sRNA) MicA has been shown to post-transcriptionally regulate translation of the outer membrane protein A (OmpA) in *Escherichia coli*. It uses an antisense mechanism to down-regulate OmpA protein synthesis and induce mRNA degradation. MicA is genomically localized between the coding regions of the *gshA* and *luxS* genes and is divergently transcribed from its neighbours. Transcription of the *luxS* gene which originates within or upstream of the MicA sequence would thus be complementary to the sRNA. LuxS regulation is as yet unclear.

**Methodology/Principal Findings:**

In this report, I show that the *luxS* mRNA exists as three long (major) transcripts of sizes that suggest just such interaction. The sRNA MicA's expression affects the abundance of each of these *luxS* transcripts. The involvement of the ribonuclease, RNase III in the accumulation of the shortest transcript is demonstrated. When MicA accumulates during growth, or is induced to be over-expressed, the cleaved mRNA species is observed to increase in intensity. Using primer extension and 5′-RACE experiments in combination with sRNA overexpression plasmids, I identify the exact origin of two of the three *luxS* transcripts, one of which is seen to result from a previously unidentified σ^S^ dependent promoter.

**Conclusions/Significance:**

The presented data provides strong evidence that MicA functions *in cis* and *in trans*, targeting both *luxS* mRNA as well as the previously established *ompA* and *phoP* regulation. The proposed *luxS* regulation by MicA would be in tandem with another sRNA CyaR, shown recently to be involved in inhibiting translation of the *luxS* mRNA. Regulation of *luxS* expression is additionally shown to occur on a transcriptional level via σ^S^ with variable transcript levels in different growth phases unlike what was previously assumed. This is the first known case of an sRNA in *E. coli* which targets both *in cis* (*luxS* mRNA) and *in trans* (*ompA* and *phoP* mRNAs).

## Introduction

Small RNAs (sRNAs) are believed to afford the bacterium an energetically cheaper, as well as a more rapid mode of affecting changes in protein production [1], [2], [3], [4]. The sRNAs thus far characterized in gram negative bacteria are overwhelmingly antisense RNAs which bind to their mRNA targets, either stimulating (rarely) or stifling (more commonly) target gene expression (see [5]). Bacterial antisense sRNAs can furthermore be categorized as either *cis*-, or *trans*-encoded, depending on the genomic localization of their targets [6]. *Trans*-encoded sRNAs are encoded from genes separate from the target encoding gene and thus complementarity between the two interacting RNAs is seldom contiguous or perfect. The RNA chaperone Hfq has been shown to be involved in accelerating the binding of some of these imperfectly binding sRNA:target pairs [7], [8]. Following binding in a subset of these transactions is often target degradation facilitated by the endoribonuclease, RNase E [9], [10], [11]. On the other hand, the *cis-*encoded sRNAs (reviewed in [12]), often found in associated genetic elements such as plasmids, phages and transposons [13] are encoded on the opposite DNA strand from their targets. They are thus perfectly complementary to their targets and are believed to be degraded in concert with their targets [14]. RNase III, a ribonuclease that recognizes double-stranded RNA (dsRNA) is known to be involved in the degradation or processing of such interacting RNAs and cuts in both antisense and target RNAs are a hallmark of such DNA duplex-dependent cleavage [15], [16], [17].

The *Escherichia coli* small RNA, MicA, is genomically localized in the intergenic region between the protein-encoding genes *ygaG* (*luxS*) and *gshA* and its function has been thoroughly elucidated in several systems [8], [18], [19]. In its best defined role, this sRNA acts as a *trans*-encoded translational inhibitor of OmpA protein synthesis [8], [18]. The *luxS* transcription start was until recently unidentified but believed to lie near the start of the *luxS* open reading frame [20], [21]. Due to this, antisense-mediated post-transcriptional regulation by MicA although postulated by [22] was yet to be shown.

This report confounds the assumption of a singular *luxS* promoter, identifying three different species of the *luxS* mRNA, two of which are apparently primary transcription products. An alluded-to transcriptional start site (within the intergenic region which ‘harbours’ MicA) is shown to also depend on the ds RNA specific endonuclease RNase III as well as the sRNA MicA; the transcript's levels increasing with MicA overexpression and decreasing with MicA depletion. I identify *in silico* as well as in vivo a stationary phase responsive (*rpoS*) promoter that drives transcription of the longest of the *luxS* species. Despite changes in relative levels of these RNAs within the system there appeared to be no effect on LuxS protein levels suggestive of an additional regulatory component. This additional component was recently identified when *luxS* translation was shown to be directly regulated by another sRNA, CyaR [23]. Combining the results of this study with those by [23], I hypothesize that MicA-mediated processing of the *luxS* mRNA modulates access of the downstream-acting CyaR to the translation initiation region (TIR). Thus CyaR would require MicA-dependent processing for it to access its target region in the *luxS* 5′-end located translation initiation (TIR) region. Interestingly, this only adds to an already emergent theme of tandem regulation found in sRNA-mediated gene regulation, albeit with a slight twist. It is the first example of a sRNA that carries out both *cis* and *trans* antisense regulation. This also further clarifies earlier suspicion of crosstalk between the outer membrane biogenesis (*ompA, phoP*) and LuxS.

## Materials and Methods

### Media and Growth conditions

Unless otherwise specified, cells were grown aerobically at pH 7.2 and 37°C in Luria broth (LB). Bacterial growth was monitored by measuring optical density at OD_600_. When required, antibiotics were added at 50–100 µg/ml (ampicillin).

### Bacterial strains and plasmids

**Figure 1:**
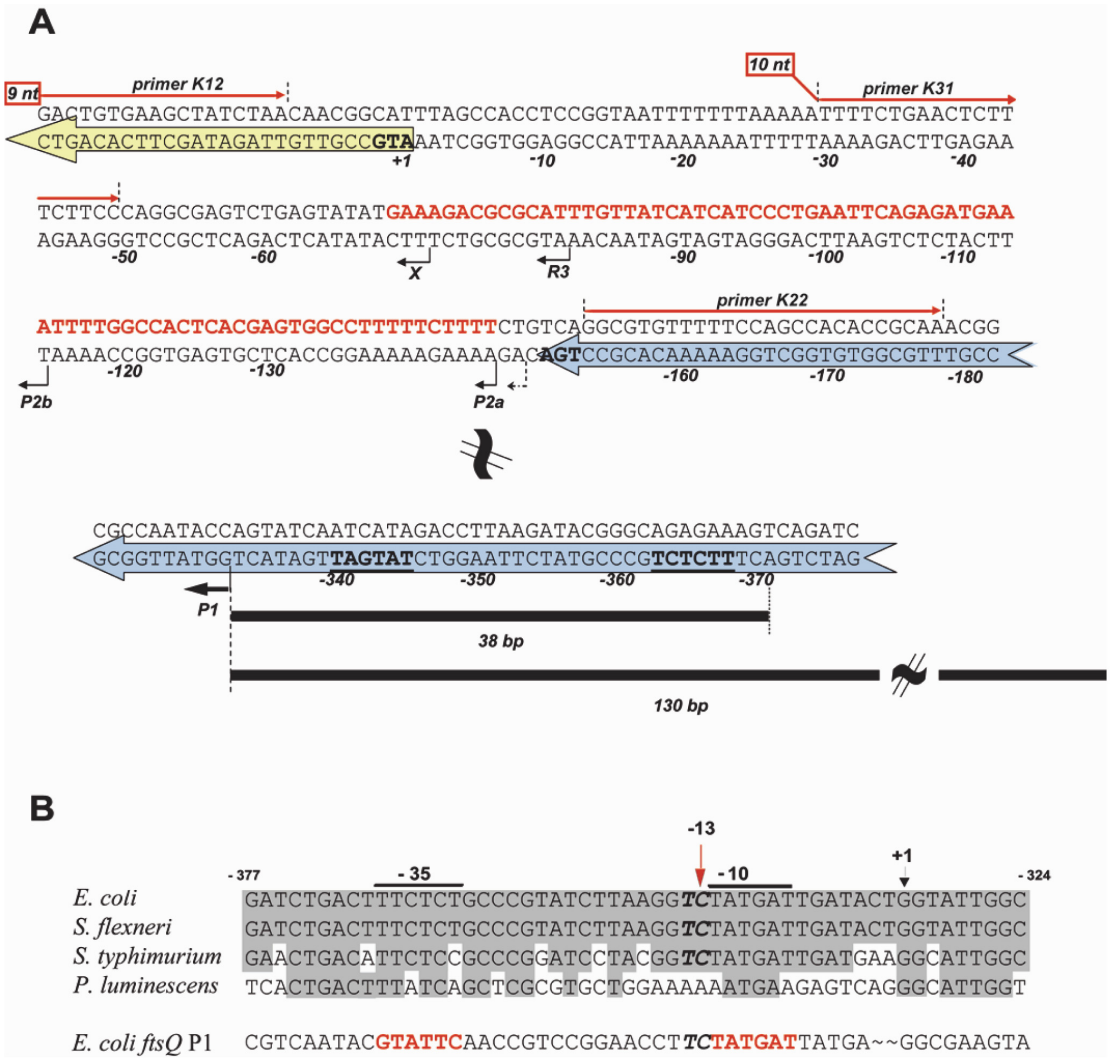
A. Schematic diagram of the genomic organization of the genes *luxS*, *micA*, and *gshA*. Arrowed bars indicate direction of *luxS* and *gshA* transcription/translation. MicA is transcribed in the opposite direction (in red) from within the intergenic region and numbering is relative to the LuxS translation start site. The relevant regions alone are shown for sake of brevity. Primer binding sites are indicated with arrows above the sequence. Short arrows beneath the sequence signify the transcript ends deduced from primer extension data (Fig. 4) and are labeled accordingly. The thick arrow labeled P1 indicates the transcript P1 as mapped by primer extension and 5'- RACE. In bold letters upstream of the P1 position are the -10 and -35 boxes. **B. Alignment of the** ***luxS*** **P1-specific promoter region.** *E. coli* sequence (bottom row panel A) was 'BLAST-aligned' against the NCBI database and the highest scoring regions from select bacteria (see text) in the genomic location *gshA_micA_luxS* were aligned against *E. coli's*. The strongly σ^S^-specific *ftsQ* P1 promoter is also included below. The RNase III-independent primer extension and 5'-RACE identified mRNA start is labeled as '+1' and indicated with an arrow. The -10 and -35 boxes are indicated by a line above the sequence alignments, the *rpoS* signatory -13 'C' lies directly outside of the -10 box and is indicated in the figure. Aligned regions correspond to equivalent positions relative to the *luxS* ORF in each species. Below the diagram are lines descriptive of the regions fused to the *luc* gene in the transcription assay (see Fig 6). (*E. coli*  =  *Escherichia coli* K12 (U00096.2); *S. typhimurium*  =  *Salmonella typhimurium* LT2 (AE008828.1); *S. flexneri*  =  *Shigella flexneri* str.301 (AE005674.1); *P. luminescens*  =  *Photorabdus luminescens* subsp. *Laumondii* (BX571863). The *E. coli ftsQ* P1 sequence is obtained from (27).

Bacterial strains and plasmids used in this study are listed in Table 1. The *E. coli* strain MC4100*relA* ^+^ was used as wildtype unless otherwise stated. To construct the transcription fusion plasmids p33_35luc, p38_41Aluc, and p40_41Aluc, the promoter region of the pZE12luc plasmid [24] was first cleaved with the restriction enzymes *EcoRI* and *XhoI*. Next, the *luxS* candidate-promoter region was amplified using primers KU33 (5′-GAA CCT CGA GCA AAT GCG CGT CTT TCA TAT; *XhoI* site underlined) and KU35 (5′-GAT AAG AAT TCG CAT TTA GCC ACC TCC GGT; *EcoRI* site underlined) for p33_35luc. For p40_41Aluc, KU40 (5′-GAA CCT CGA GCT TTC TCT GCC CGT ATC TTA; *XhoI* site underlined) and KU41A (5′-GGT GAG AAT TCC AGT ATC AAT CAT AGA CCT; *EcoRI* site underlined) were used and for p38_41Aluc, KU38 (5′-GAA CCT CGA GGT CGC GCA AAC GCT GGA TAG TA; *XhoI* site underlined) and KU41A above. Fragment sizes were 110 bp, 62 bp, and 163 bp respectively. The amplified regions around P1 are depicted schematically in Figure 1A. All PCR fragments were linearized in the same manner as the vector and ligation was carried out using T4 DNA ligase according to standard protocol following agarose gel purification of the DNA fragments. The resultant plasmid was thus comprised of the *luciferase* gene under transcriptional control of the putative *luxS* promoter region. The luminescence background control plasmid (pZE12b_EX) bearing a promoterless *luc* gene was constructed as previously described [25]. Strains were transformed using standard molecular biology protocols.

**Table 1:** Strains and plasmids.

Strain name	Bacteria	Genotype	Reference
MC4100 *relA*+	*Escherichia coli*	*araD*139 (argF-lac)205, *flb*-5301, *pstF*25, *rpsL*150, *deoC*1	T. Nyström
MC4100 *relA*+ RNase III*-*	*Escherichia coli*	*araD*139 (argF-lac)205, *flb*-5301, *pstF*25, *rpsL*150, *deoC*1, *rncA*14	G.Wagner

Plasmid trivial name	Plasmid name	Properties	Reference
pMicA	pJV150IG-34	pZE12-*luc* derivative (ColE1, Amp^R^), *P*_LlacO_ promoter, *micA* insert	Udekwu *et al.* 2005
pAntiMicA	pJV721-2	Reverse *micA* insert followed by *rrnB* terminator in pZE12-luc	Udekwu *et al.* 2005
pControl	pJV968-1	Promoterless *lacZ* fragment inserted in pZE12-*luc* as above	Vogel *et al*. 2004
pZE12-*luc*	pZE12-*luc*	p15A plasmid with pLac-*luc*	Lutz & Bujard. 1997
pZE12b-EX	pZE12b-*luc*	ColE1, Amp^R^ plasmid with promoterless *luc* gene	Udekwu & Wagner. 2007
p33_35*luc*	p33_35*luc*	*luxS* -84 → +6 cloned upstream of *luc* gene in pZE12-*luc* *	This study
p38_41A*luc*	p38_41A*luc*	*luxS* -462 → -332 cloned upstream of *luc* gene in pZE12-*luc* *	This study
p40_41A*luc*	p40_41A*luc*	*luxS* -371 → -332 cloned upstream of *luc* gene in pZE12-*luc* *	This study

*Numbering relative to *luxS* translation start site.

### Northern blot analyses

Cells were harvested at specific points during growth by quenching in 0.2 volumes of RNA stop solution (5% phenol, 95% ethanol). These were centrifuged and pellets snap-frozen in liquid nitrogen. Total RNA was extracted using the hot acid-phenol method essentially as described [15]. Total RNA was treated with RQ1 DNase (Promega), extracted with phenol, then chloroform, and precipitated in ethanol at –20°C. The RNA was pelleted at 4°C, washed with 75% ethanol, dried at room temperature and resuspended in sterile RNAse-free water. One volume of RNA loading buffer (95% [v/v] formamide, 0.025% [w/v] bromophenol blue, 0.025% [w/v] xylene cyanol, 0.025% [w/v] SDS, 5 mM EDTA pH 8.0) was added. Electrophoresis of total RNA was carried out under denaturing conditions, on 5% or 8% polyacrylamide gels containing 7 M urea. Gels were electroblotted (Bio-Rad Trans-Blot cell) onto Nylon N+ membranes (GE Healthcare) and probed in modified Church and Gilbert hybridization buffer [26]. Probing with DNA oligodeoxyribonucleotides was carried out at 42°C or with riboprobes at 65°C. Hybridized probes were visualized with a PhosphorImager, model 400S (Molecular Dynamics), and band intensities quantitated using ImageQuant software, version 4.2a (Molecular Dynamics).

### Promoter sequence alignment

Sequences obtained from the BLAST database (http://www.ncbi.nlm.nih.gov/BLAST)corresponding to the homologous *gshA_luxS* coding regions from several close relatives of *E. coli* were manually aligned. The sequences within the coding region of *gshA* that correspond to the *luxS* P1 region of *E. coli* were identified and the orientation of genes in this genomic region checked for conservation [18]. The alignment was carried out on four different homologous sequences of ranging depth within the gamma proteobacteriaceae subclass. Genomic sequence spanning –355 to –301 relative to the *luxS* translation start site of *E. coli* and corresponding regions of three closely related species were aligned against each other (Figure 1B). All BLAST sequence ID s are listed in the figure legend and the *ftsQ* p1 promoter is described in [27].

### Riboprobe generation

Radioactive DNA probes were generated by 5′-end labelling of oligo-deoxyribonucleotides complementary to the RNA, with a molar excess of γ-^32^P-ATP. The *luxS* riboprobe was generated by hot *in-vitro* transcription essentially as described previously [18] using the oligonucleotides T7-luxSRPend (5′-GGT AAT ACG ACT CAC TAT AGC TAG ATG TGC AGT TCC TGC AAC T) and 3′luxS RP (5′-ATG CCG TTG TTA GAT AGC TTC ACA). Purification of probes was carried out by passing them through G50 Microspin columns (GE Healthcare).

### Transcription assay

Aliquots of 1 ml each were taken from growing cells and quenched in chloramphenicol (200 µg/ml final concentration) prior to snap freezing in liquid nitrogen. Samples were lysed and assayed for luciferase activity using the Luciferase Assay Kit (SIGMA), according to the manufacturer's protocol, on a Bio-orbit 1253 luminometer (Bio-orbit Oy). Background luminescence was obtained from cells carrying the transcriptionally inactive control plasmid (pZE12b_EX).

### Primer extension

Primer extension was carried out using Superscript II reverse transcriptase, on 10 µg of total RNA using oligonucleotide primers K12 (5′-GGT ATG ATC GAC TGT GAA GCT ATC TAA) or K22 (5′- GGC GTG TTT TTC CAG CCA CAC CGC AA), 5′-end labeled with γ ^32^P-ATP (GE Healthcare) as described earlier. Probes were purified on denaturing 15% PA gels and eluted with RNA elution buffer [0.1 M Sodium acetate (pH 5.7), 10 mM EDTA, 0.5% SDS]. After overnight elution, probes were phenol-chloroform extracted and precipitated in ethanol for 1 h at −20°C prior to use. Extension was typically for 40 min at 55°C, and RNA was hydrolyzed by addition of 1/3 volume (v:v) of 3 M KOH, followed by heating to 95°C for 5 min. After this, the cDNA was precipitated in 3 vol of ethanol and finally resuspended in 15 µL of loading buffer II (Ambion). Electrophoretic analysis was carried out on 8% polyacrylamide gels containing 7 M urea.

### 5′- Rapid amplification of cDNA ends (5′-RACE)

5′-RACE was carried out on 18 µg of total RNA essentially as described [28], except for minor modifications. 5′ triphosphates were converted to monophosphates by treatment of 15 µg total RNA with 25 units of tobacco acid pyrophosphatase, TAP (Epicentre Technologies). The reaction was carried out at 37°C for 60 min in a total reaction volume of 50 µl containing 50 mM sodium acetate (pH 6.0), 10 mM EDTA, 1% β-mercapto-ethanol, and 0.1% Triton X-100. Control RNA was incubated under the same conditions in the absence of enzyme. Reactions were stopped by phenol/chloroform extraction, followed by ethanol precipitation. Precipitated RNAs were re-dissolved in water, mixed with 500 pmol of the 5′–end RNA adapter, A3 (5′-GAU AUG CGC GAA UUC CUG UAG AAC GAA CAC UAG AAG AAA: Dharmacon Research), heat-denatured at 95°C for 5 min, then snap-cooled on ice. Adapter ligation was carried out at 16°C for 12 hr with 50 units of T4 RNA ligase (New England Biolabs) in a buffer containing 50 mM Tris-HCl (pH 7.9), 10 mM MgCl_2_, 4 mM DTT, 150 µM ATP, and 10% DMSO. Phenol/chloroform-extracted, ethanol-precipitated RNA (∼9 µg) was reverse-transcribed using 2 pmol of the *luxS*-specific primer K12 (see above) and Superscript II reverse transcriptase (Invitrogen) according to the manufacturer's instructions. Reverse transcription was performed in three subsequent 20 min steps at 55°C, 60°C, and 65°C and concluded by RNaseH treatment. The reverse transcription products were amplified using a 1 µl aliquot of the RT reaction and 25 pmol of cloning primer K31 (5′- GAA CCT CGA CTT TTC TGA ACT CTT TCT TCC ) and B6 (5′-ACG ACG TTG TAA AAC GAC GG). The underlined sequence in K31 is complementary to the *luxS* upstream region. In order to map the end of band P1, I used primer K22 and the same B6 primer above. Standard PCR amplification was carried out and products were separated on 2% agarose gels, bands excised and gel-eluted using the QIAgen gel extraction kit (QIAGEN). The extracted DNA was cloned into the pCR 2.1 TOPO vector (Invitrogen) and transformed into TOPO TA competent cells according to the manufacturer's protocol. At least 10 colonies per cloned gel fragment-carrying insertions were sequenced using primer FP0519 (5′- CTT TAT GCT TCC GGC TCG TAT G) and RNase inhibitor (Ambion) supplemented all enzymatic reactions carried out on RNA.

## Results

### Analysis of *luxS* mRNA species *in vivo* and determination of 5′-ends

#### Northern blot analysis of *luxS* mRNA in wild-type and RNase III mutant strains

**Figure 2:**
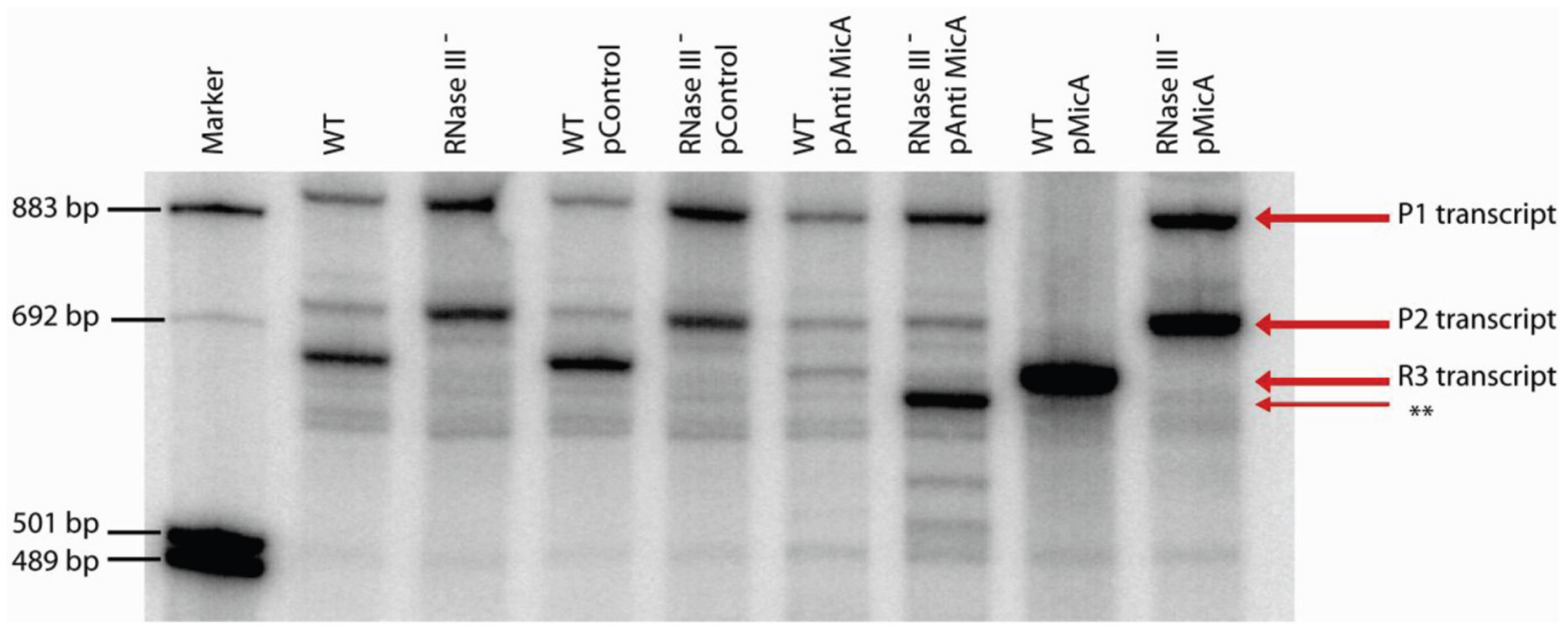
Northern blot analysis of *luxS* mRNA steady state levels in wildtype (lanes 2, 4, 6 & 8) and an isogenic *rnc* ^-^ mutant strain (lanes 3,5,7 & 9). The strains carried either no plasmid (lanes 2 & 3); control plasmid (lanes 4 & 5); AntiMicA overexpressing (lanes 6 & 7); MicA overexpressing (lanes 8 & 9). RNA was extracted in stationary phase and 10 μg of total RNA was analyzed on 5% PA gels prior to transfer to charged nylon membranes. Probing was carried out with an in vitro synthesized luxS riboprobe (LuxS RP). The different RNA species are indicated in the figure. Equal loading was ensured by probing and normalizing to 5S RNA.

Previous studies carried out on *luxS* did not shed light on this gene's transcriptional regulation and with a dearth of strong transcription signals in the region immediately proximal to the *luxS* coding sequence I opted to probe for this mRNA on a northern blot. I inferred transcription through the intergenic region due to the unmapped status of *luxS* transcription. For this reason, plasmid-containing wildtype and RNase III minus *E. coli* (constitutively overexpressing MicA or AntiMicA) were examined. Total RNA was separated on a denaturing polyacrylamide gel, transferred onto a nylon membrane and probed for *luxS* mRNA with a riboprobe spanning the entire coding region. Multiple species (three distinct bands) of *luxS* mRNA were detected in both backgrounds. The detected transcripts were denoted P1, P2, and R3 as seen in Figure 2. An additional (weak) band was also observable and this was strongly enhanced in the RNase III minus background with AntiMicA overexpression [‘**’ in Fig. 2]. Steady-state levels of the *luxS* P1 transcript when in stationary phase (OD_600_  = 2.5) are higher than P2 in a wildtype setting (Fig. 2, lanes 2 & 4). Upon MicA overexpression, the R3 transcript is seen to accumulate as a reciprocal decrease in P1 and P2 RNA is observed (Fig. 2, lane 8). To add to this, R3 was not observed under any conditions in the RNase III-deficient strain (Fig. 2, lanes 3, 5, 7 and 9)and the P1 and P2 band intensities were both elevated compared to wildtype in this background (cv Fig. 2, lane 3 vs 2; lane 5 vs 4). Taken together, this strongly suggests that R3 is processed from P1 and P2 in an RNase III-, and MicA – dependent manner. To support this, when AntiMicA RNA is overexpressed in a wildtype background, R3 accumulation is >70% reduced compared to control (cv Fig. 2 lane 6 vs lane 4) although Anti-MicA overexpression is only about 80% efficient in titrating out MicA [18].

#### Northern blot analysis of transcript abundance during *in vitro* growth in LB broth

**Figure 3:**
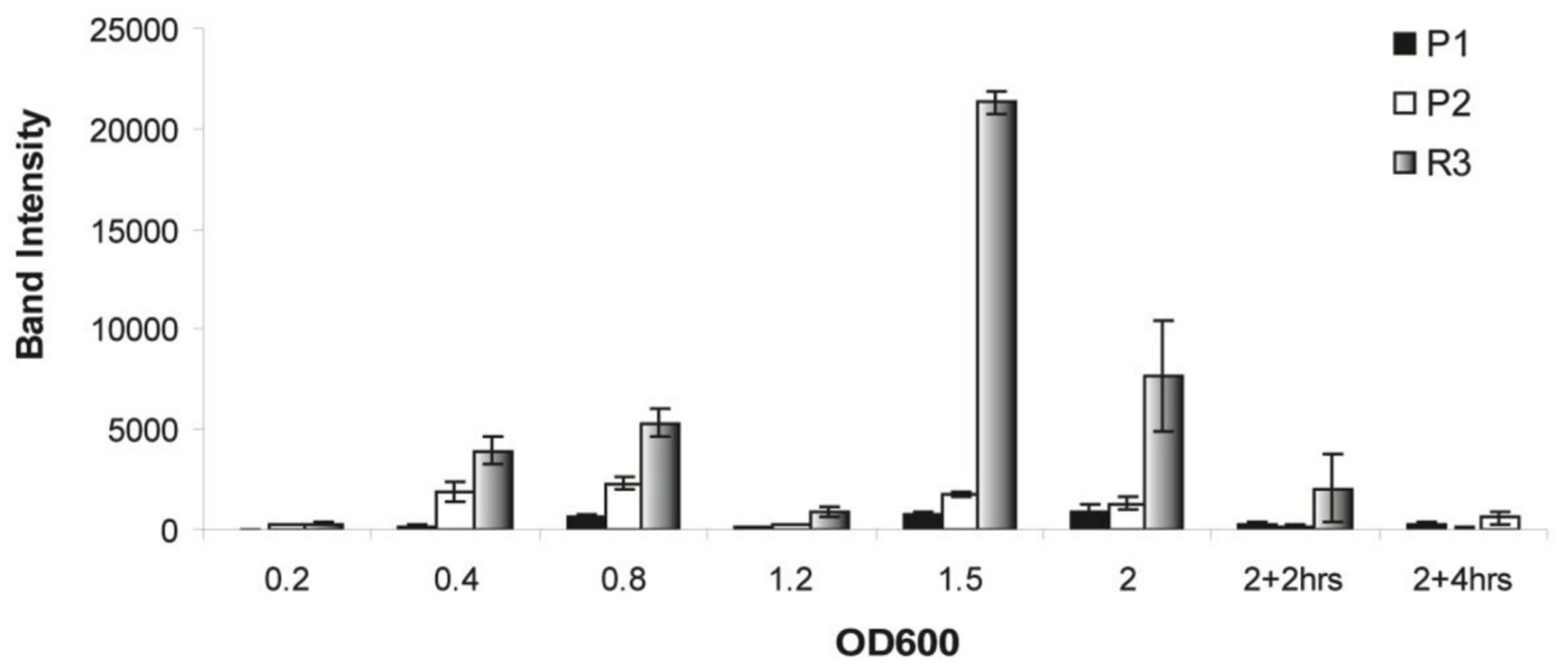
Steady state levels of *luxS* mRNA transcripts during growth in liquid culture. Intensity of *luxS* probing-generated transcripts P1(black) and P2 (open), as well as R3 (shaded), at various stages of growth as assayed by Northern Blot analysis. Signal intensities as derived from band densitometry were normalized to 5S rRNA levels and plotted accordingly.

Figure 3 shows quantitated band intensities during growth, for the P1, P2, and R3 RNA species. All observed RNA species increased in intensity during growth, with peak levels evident at OD600 ∼0.8 for P2, and OD600 ∼1.5 for P1 (Fig. 3, open and filled bars, respectively). The transcript denoted as R3 increased in abundance through growth in a manner similar to the observed expression profile of the sRNA MicA [25]; it increases monotonically, peaking in mid stationary phase prior to a reduction in transcript intensity through late stationary phase.

### Primer extension analysis

**Figure 4:**
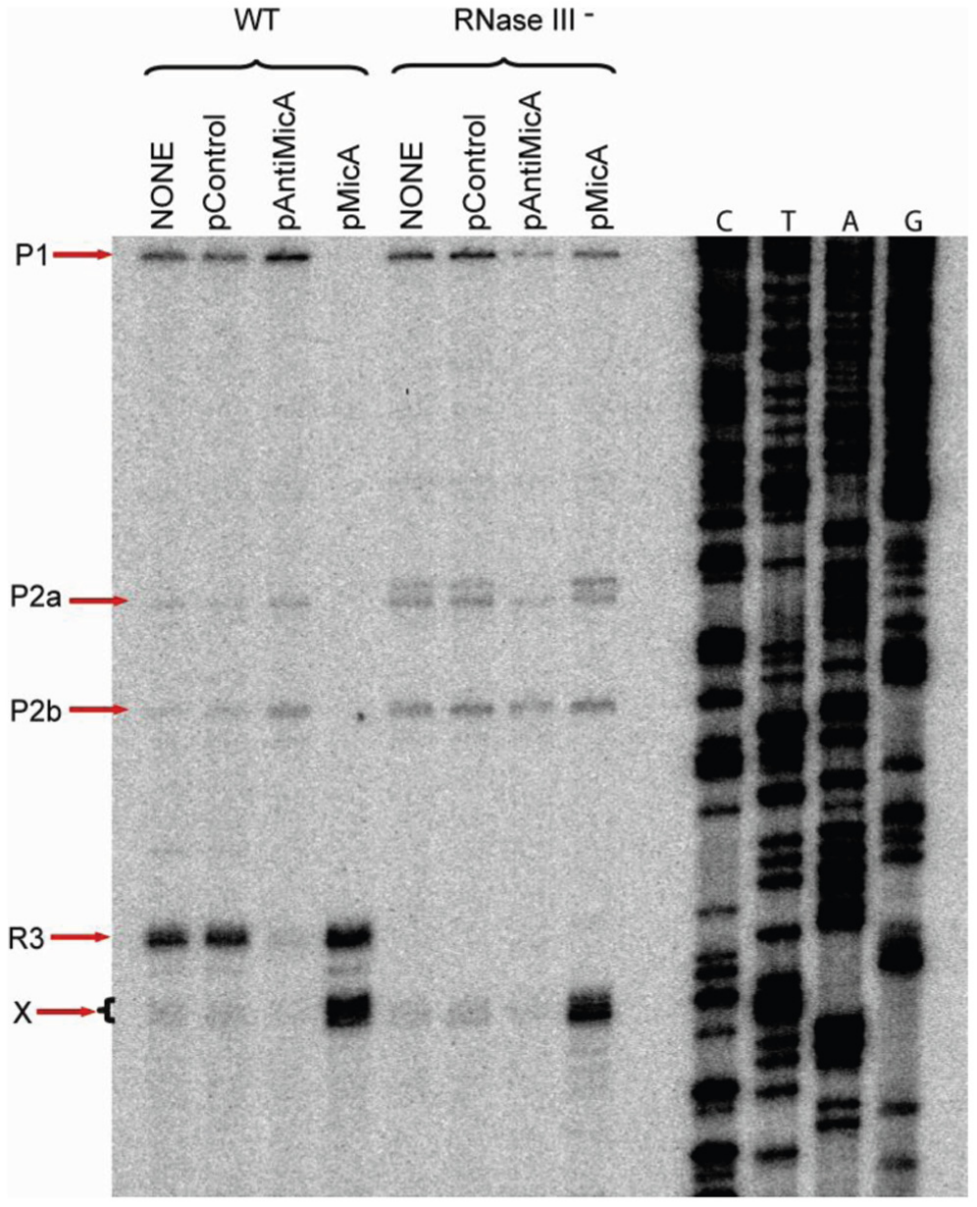
Primer extension assay carried out on total RNA extracted from cells grown to stationary phase. ^32^P 5'-end labelled K12 primer was extended with the Superscript II reverse transcriptase and resolved on 7% polyacrylamide gel. Major bands are delineated in the schematic of figure 1. P1 represents the longest transcript (> 800 nt); P2 is somewhat shorter (> 650 nt); R3 (∼ 600). An additional band of unexplained origin, X, is also indicated in the figure. Band 2a is seen to be accompanied by an additional unlabelled band in the *rnc* ^-^ mutant.

To broach the question of the multiple *luxS* mRNA bands, primer extension analysis was used to identify 5′-end heterogeneity. Extending a primer (K12), complementary to the 5′-end of the *luxS* coding region for primer extension analysis, the different ends of the *luxS* transcripts were identified. This was carried out on RNA extracted from stationary phase (OD_600_ ∼3) cultures of the same *E. coli* strains in the preceding experiment. To accurately identify the longer P1 transcript, another primer (K22) complementary to the 3′-tail of the *gshA* mRNA was used (gel not shown). All the mapped 5′-ends evident in Figure 4 are indicated in the schematics of Figure 1. In summary of those results, the P1 and P2 (a,b) bands disappear specifically under MicA overexpression (Fig. 4, lane 4 vs lanes 1 and 2). This corroborates the MicA dependence of processed band R3 observed in Figure 2. Also in line with the Northern blot data, AntiMicA overexpression in a wild-type background leads to a strongly reduced band intensity of R3, which is absent in the RNase III (-) strain (lanes 5 to 8). This happens probably due to the presence of AntiMicA which would act as a decoy target for MicA. These data taken together strongly suggest that; (a) the heterogeneity of *luxS* mRNA length is as a result of variation in the 5′-ends of the RNA, (b) *luxS* is transcribed from at least one promoter, found >300 bp upstream of its coding sequence, and (c), that one of the three identified *luxS* specific RNA species (R3) is MicA- and RNase III-dependent.

### 5′-RACE analysis

**Figure 5:**
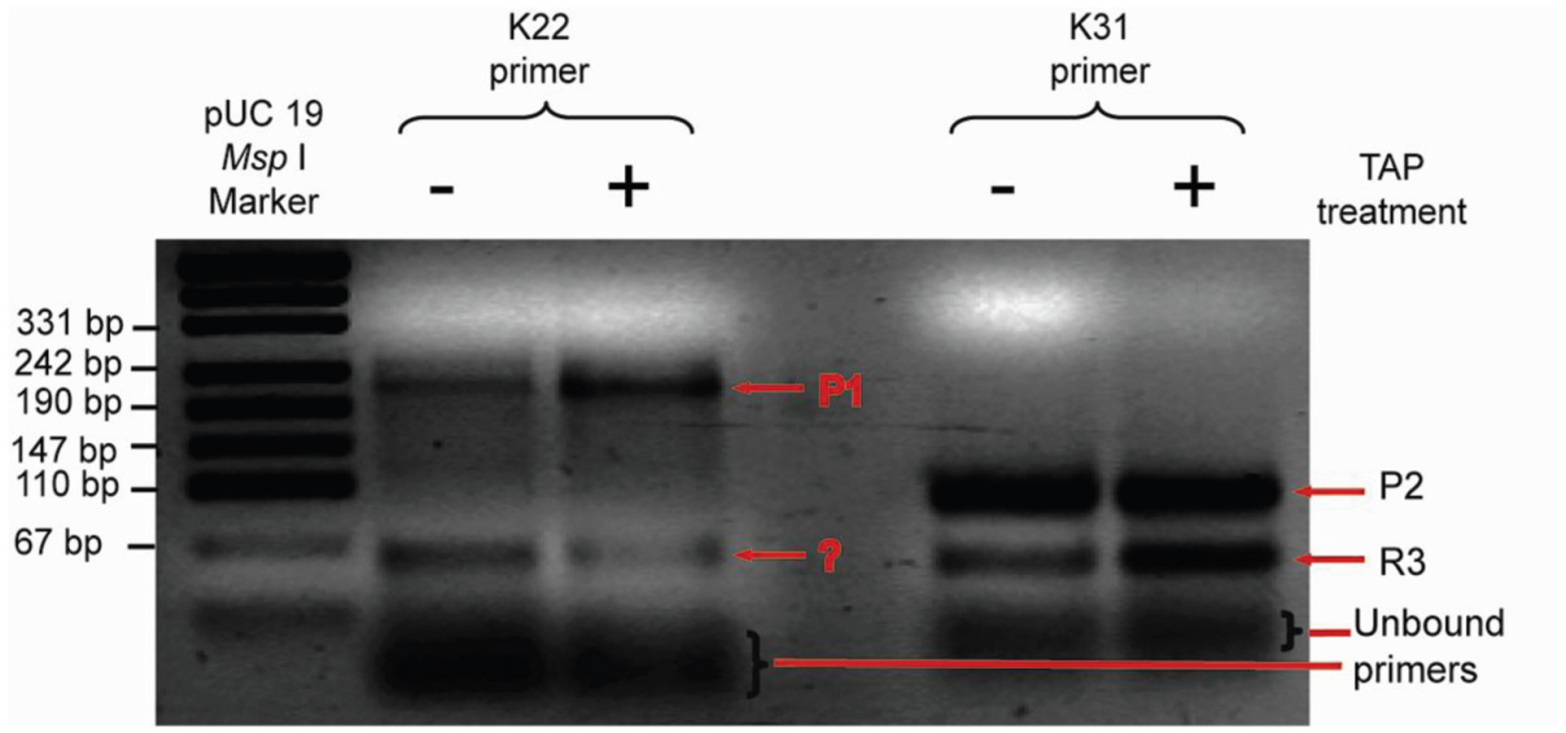
5'- RACE amplified products of first strand synthesized products using the adapter specific B6 primer in combination with the K22 primer which binds to the *gshA* terminus, or K31 which is complementary to the sequence spanning 31 bp to 50 bp upstream of the *luxS* ORF. Resultant PCR products were resolved on 2% agarose gels against pUC *MspI* marker (Fermentas). TAP  =  tobacco acid pyrophosphatase. The P1-generated and TAP sensitive band was cloned into pTOPO 2.1 plasmids and resultant clones sequenced to give the indicated G (-332) as transcription start site.

To further characterize the observed transcripts as primary or processed such, 5′-RACE analysis was carried out on untreated or tobacco acid pyrophosphatase (TAP)-treated *E. coli* total RNA. TAP-treatment being specific for triphosphorylated 5′-ends serves to enhance signal from primary transcription products. Figure 5 shows a 2.0% agarose gel analysis of 5′RACE products generated by primers K22 or K31, and E6 (*luxS* - leader specific and adapter-specific, respectively). These products upon sequencing (7 of 10 clones) identified a TAP-enhanced end as a ‘G’ residue at position −332 relative to the *luxS* AUG (translation) start which gave rise to band P1 (Fig. 5, Lane 3). The remaining three clones were found to be around 30 bases shorter (−298, −291, −284) although no promoter-like sequences are evident in the vicinity of these cloned ends. The R3 band (Lane 6) is somewhat perplexingly also enhanced by TAP, implying that it too is a primary transcript in line with observations by [23]. Strikingly, this RNase III-dependent end (this study) maps to a transcription start site for this mRNA and although not currently understood, is observed with the *rpsO* 5′-UTR of *Streptomyces antibioticus* [29]. Clones that clarified to some extent (see Discussion) the nature of the P2a and P2b bands from the primer extension assay were found within MicA in areas of high AU-richness. Combining their low cloning frequency with the AU-richness of the region, I tentatively ascribe these ends to ss endoribonuclease-mediated cleavage of the *luxS* mRNA.

### Sequence alignment

I aligned the corresponding promoter (P1 above) region from three related gram (-) bacteria one of them being the obligate symbiont, *Photorhabdus luminescens*, recently shown to have the same genomic organization [30]. The region immediately upstream of the experimentally identified +1 site of the P1 RNA in *E. coli* shows characteristics of an σ^S^-responsive gene. An extended −10 box matching exactly that of the highly σ^S^-specific *ftsQ* P1 promoter and a conserved ‘C’-residue is present just upstream at the predicted −13 position (indicated in Fig. 1B). The conservation of the upstream region is very strong between *E. coli* and its closest relatives *Salmonella typhimurium* and *Shigella flexneri.* This complementarity continues to *P. luminescens*, primarily in the −35 and −10 boxes where 4 of 6 residues in each box (67%) are absolutely conserved, compared to the surrounding region (35%).

### Transcriptional fusions confirm promoter functionality

**Figure 6:**
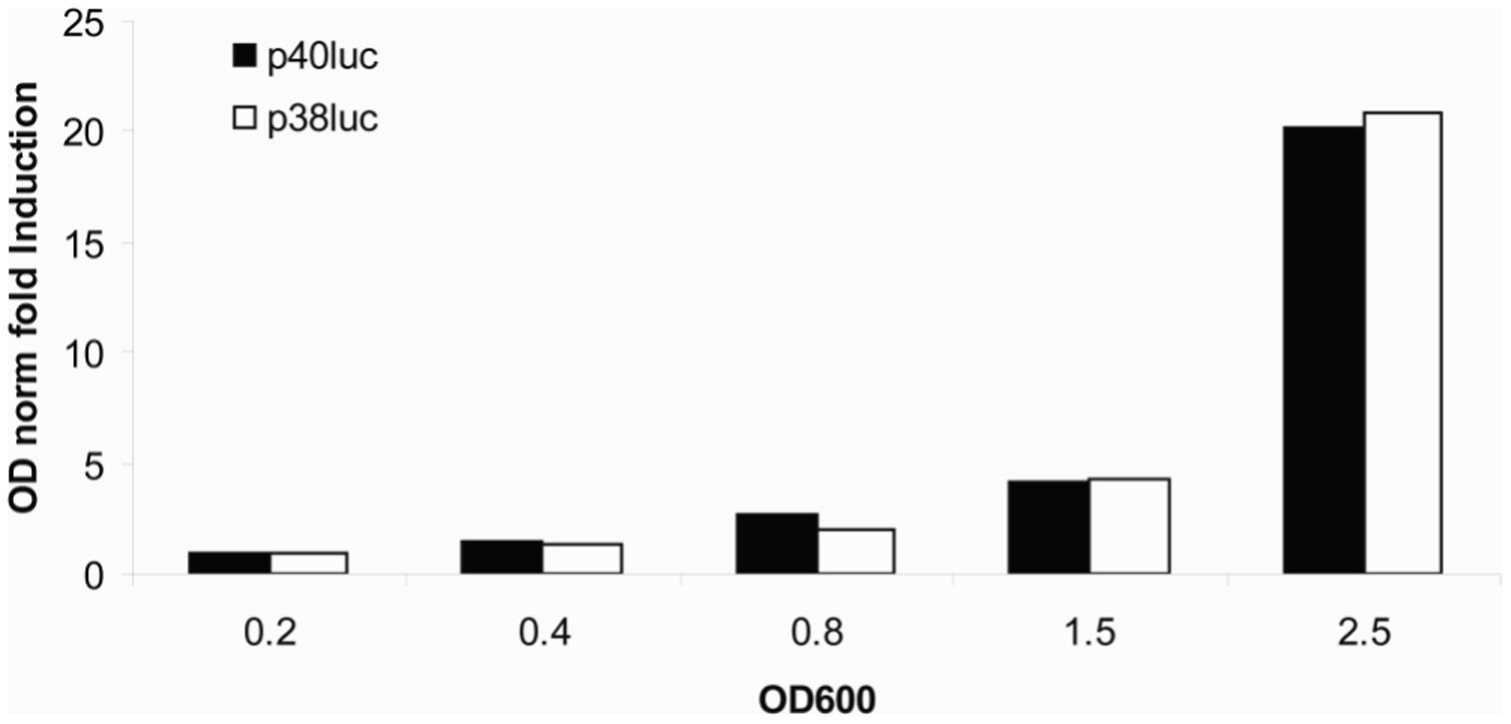
Transcriptional fusions to a *luc* reporter gene of two different lengths of the putative *luxS* promoter pP1 assayed for activity. The long fusion p38 (open bars) encompasses 130bp of sequence around the putative promoter whilst the shorter p40 (filled bars) is 38 bp long encompassing only the -35 and -10 boxes. Cells were grown in LB medium until stationary phase, with aliquots taken at various OD_600_ as indicated in the figure. A transcriptionally inactive plasmid was used to normalize for background activity. All values are OD normalized and plotted as fold induction over values at the earliest timepoint.

To test for activity of the putative promoter P1 *in vivo*, I introduced the candidate P1 sequence (of different sizes) upstream of a promoterless luciferase gene carried on a plasmid. Compared to the promoterless (p33_35luc) plasmid (control for background luciferase activity), high transcription activity was observed from the P1 promoter. This activity was seen to increase upon entry into stationary phase (Fig. 6). The results showed that 130 bp of sequence upstream of P1 drove the transcription of luciferase efficiently (Fig. 6, white bars). Even a truncated construct containing the 38 bp directly upstream of P1 displayed this activity (Fig. 6, black bars). When I instead inserted the proximal 70 bp upstream of the *luxS* coding region upstream of the *luc* gene, no significant promoter activity was detectable (data not shown). In addition to this, the p40_41A construct showed little activity in an rpoS (-) background (data not shown). This data suggests strongly that the *luxS* P1 transcriptis transcribed from a coding region-distal promoter in an rpoS dependent manner.

## Discussion

LuxS catalyzes the breakdown of S-ribosylhomocysteine or SRH to 4,5-dihydroxy-2,3-pentanedione, or DPD and homocysteine [20], [31]. DPD is a precursor of the autoinducer 2 molecule (AI-2), and AI-2 has been proposed to be a global quorum sensing signal [21], [32]. Despite the essentiality of LuxS for AI-2 production, it has been claimed that Pfs, the upstream enzyme in this pathway, is rate-limiting for AI-2 production in *E. coli* [20]. This was based on the transcriptional activity of a fusion constructed on the assumption of a *luxS* transcription start that was not experimentally supported [21]. In my hands, at least the transcription of the *luxS* mRNA but not the protein is under growth phase regulation, most likely involving σ^S^.

This study describes RNase III- and MicA dependence of *luxS* mRNA abundance(s) while identifying a new *luxS* promoter. The complex transcriptional architecture of the *luxS*-MicA-*gshA* region of *E. coli* is described with potential translational relevance of the interaction between MicA and *luxS* mRNAs. When studies commenced on MicA, two functions of this sRNA were considered: 1) MicA could act as a regulator of *trans-*encoded mRNAs, or 2) MicA could represent a *cis-*encoded antisense RNA, provided that either the 3′ UTR of *gshA*, or the 5′UTR of *luxS* mRNA overlap the sRNA gene sequence (See Fig. 1A). Naively, at the time a single target was assumed. Although *gshA* and *luxS* are transcribed in the same orientation relative to each other, there is no evidence of a polycistronic message containing both reading frames in *E. coli* [20]. Bacterial gene regulation in the 5′-regions of mRNAs is substantially more widespread and as long 3′-UTR's are unusual in bacteria, the differentially sized *luxS* riboprobe-specific mRNAs were suspected to vary on the 5′-end. Transcription of *luxS* mRNA was reported to be from a singular promoter which was not directly identified [33]. The recent identification of a TAP-dependent singular transcript corresponding to our R3 transcript unfortunately did not identify a promoter sequence however [23]. It was also widely accepted, though without convincing evidence, that *luxS* is not differentially regulated on the transcriptional level in gram (-) bacteria [21]. This had struck me as unusual as the gene product has been shown to be essential for the stationary phase abundant AI-2 synthesis in *E. coli* [33]. However, inconsistent with this, *luxS* is known to be differentially regulated, and even transcriptionally so in *Edwardsiella tarda* [34].

Importantly, three major *luxS* - specific bands were observed in a Northern blot analysis (Fig. 3). At least two of three bands were of a size corresponding to transcripts originating from beyond MicA [35]. One band in particular was of a size that suggested a 5′- end at least 300 nt upstream of the *luxS* start codon. It became obvious, based on band-length, that the region used previously in *luxS* - *lacZ* transcriptional fusions [21] could not account for the most slowly migrating band (P1). Simultaneously, 5′- end heterogeneity would imply transcription overlapping the intergenically located MicA RNA for both mRNA species (Bands P1 and P2). Both of these longer transcripts could theoretically interact with the full-length MicA RNA although the third band would only overlap by about one helical turn (14nts in this study, 15 nts in [23]) precluding RNase III processing; RNA duplexes formed are targets for RNase III [17] which specifically recognizes and cleaves double-stranded RNA of at least two helical turns [36]. There is more than sufficient transcription overlap (*luxS* transcription proceeds through the MicA encoding region) to justify probing for *luxS* mRNA in an RNase III deficient mutant (*rnc* ^-^). Notably also, the consensus-observed RNase III cleavage sequences are quite similar to the expected region of interaction between the MicA 5′-end and its complimentary *luxS* upstream region (data not shown). Primer extension specifically identified the P1 product in Figure 4 although its exact end was deduced by RACE analysis using a more ORF-distal primer, K22. The cloning of primarily (7/10) fragments identifying the same G residue (−332) as the transcription start in 5′-RACE, was consistent with primer extension data. The P2 products were evident only when K31 was used as K22 binds downstream of the primer extension predicted ends (shown in Fig. 1A). Despite not describing the P2 band as extensively as P1, the primer extension derived 5′- ends which were TAP-independent (Fig. 1A) implied its promoter (if a primary transcript) would be located in the most distal 3′- end of the *gshA* message. However, the nature of the region where these transcript 5′- ends are found, combined with their poor representation among the clones in my RACE analysis is reminiscent more of endonuclease processed RNAs. All this and the band intensity reduction of P1 and P2 transcripts is still consistent with RNase III processing should interaction occur with MicA and as its steady state levels increase during growth (See Fig. 3). In fact, the R3 product follows an accumulation profile during growth that is quite reminiscent of that of MicA ([18]; K.U., data not shown).

Further evidence of MicA involvement in R3 accumulation stems from the increased presence of the processed R3 band and the concomitant decrease in both P1 and P2 levels during growth (Fig. 2, OD_600_ 0.2 to 1.5), and upon overexpression of MicA (Fig. 3, lane 8 vs lane 2 & 4). This is not the case in a Δ *rnc* strain where the R3 species is completely absent (Fig. 3, lanes 3, 5, 7 & 9). Another band denoted ‘X’ (mapping to position +4 of MicA as shown in Fig. 1) is seen in the primer extension experiment to increase in intensity when MicA is overexpressed in both strains but it remains unclear what the nature of that particular band is. After identifying the 5′-end of the P1 RNA, it was established that transcription occurs *in vivo* from the relevant sequence upstream of the P1 start. Transcriptional fusions of this region (see Fig. 1) displayed high activity which increased (∼10 fold) upon entry into stationary phase (Fig. 6). Thus, the P1 transcript would correspond to the slowest migrating band observed in the Northern blot analysis of *luxS* expression.

There is somewhat of an impasse with regards to the ‘true’ function of the LuxS protein in *E. coli* and its close relatives. Briefly, some researchers are of the opinion that AI-2 is primarily a metabolic bi-product [37] while others focus on a role as a universal signaling molecule [38]. Inadvertently with LuxS regulation in focus, this study has been on the above described transcription of the *luxS* gene and its processing by MicA. Although Wang et al (2005) observed a negative regulation of *luxS* expression by cAMP and CRP, an alternative explanation is MicA involvement as it is up-regulated in a *cya*-deficient background [25]. All our data suggest strongly that in *E. coli* and perhaps close relatives of it, *luxS* is not only translationally but also transcriptionally (this work and [39]) and post-transcriptionally (this work, [19], [23]) regulated during growth. The data presented is also supported by a recent publication, where variable *luxS* gene expression in *E. coli* was documented albeit on a protein level [40]. In this proteomics-based mapping of the newly - elucidated Crl/σ^S^ regulon, the authors reported ∼5 fold lower LuxS protein levels in an *rpoS* mutant strain compared to wildtype. They additionally show a 20 fold lower expression level in an *rpoS*, *crl* double mutant. This observation is entirely in line with the results presented here; *luxS* mRNA is upregulated in stationary phase and the P1 transcript displays characteristics of being σ^S^-specific. Lelong *et al.* [40] show the existence of ‘stationary phase regulatable’ protein levels at the very least. Although uncertain whether or not the changes that they see are as a result of increased transcription, the effect of MicA-mediated processing is currently being addressed. The identification of the *luxS* mRNA as an additional target for MicA RNA is intriguing in any case. At this point of investigation, the MicA/RNAse III-dependent processing of the longer *luxS* transcripts to the R3 transcript is strongly suggestive of sRNA-mediated regulation. Arguably, any effect of MicA on transcript abundance (shown) or stability (also shown) is gene-regulatory in nature. However, the effect of overexpressing MicA on LuxS protein levels was not evident; immunoprecipitated, radiolabelled, pulse-chased LuxS protein did not differ significantly between induced and non-induced MicA samples (data not shown). If MicA were to affect the synthesis of the LuxS protein, then it could be suggested that this sRNA would act as an interface of sorts between quorum sensing and membrane sensing. De Lay and Gottesman described the small RNA (CyaR), showing strong evidence for LuxS translation regulation. However, unlike their data, we observed 3 isoforms of the *luxS* mRNA (primarily due to higher resolution of polyacrylamide gels). Although this study does not contradict their results regarding the regulation by CyaR, it does complicate the regulatory circuit somewhat. MicA is constitutively up-regulated in a cyclic AMP deficient strain (*cyaA* ^-^)[25] and this would accordingly increase the abundance of the *luxS* P1 transcript. In combination with my data, it appears that the post-transcriptional regulation of LuxS protein levels could feasibly be dependent on the two sRNAs (MicA and CyaR) as well as two RNases (RNase III and RNase E respectively). It still remains to be tested however, whether or not MicA-dependent processing of the *luxS* mRNA is essential for CyaR-mediated regulation or if the process can be by-passed. Work is ongoing to clarify the putative tandem activity of these coordinate sRNAs.

## References

[1] Mitarai N, Benjamin JA, Krishna S, Semsey S, Csiszovszki Z (2009). Dynamic features of gene expression control by small regulatory RNAs.. Proc Natl Acad Sci U S A.

[2] Mitarai N, Andersson AM, Krishna S, Semsey S, Sneppen K (2007). Efficient degradation and expression prioritization with small RNAs.. Phys Biol.

[3] Montzka Wassarman K, Zhang A, Storz G (1999). Small RNAs in Escherichia coli.. Trends in Microbiology.

[4] Vogel J, Wagner EG (2007). Target identification of small noncoding RNAs in bacteria.. Curr Opin Microbiol.

[5] Gottesman S (2005). Micros for microbes: non-coding regulatory RNAs in bacteria.. Trends in Genetics.

[6] Wagner EGH, Flardh K (2002). Antisense RNAs everywhere?. Trends in Genetics.

[7] Chen S, Zhang A, Blyn LB, Storz G (2004). MicC, a second small-RNA regulator of Omp protein expression in Escherichia coli.. J Bacteriol.

[8] Rasmussen AA, Eriksen M, Gilany K, Udesen C, Franch T (2005). Regulation of *ompA* mRNA stability: the role of a small regulatory RNA in growth phase-dependent control.. Molecular Microbiology.

[9] Kawamoto H, Koide Y, Morita T, Aiba H (2006). Base-pairing requirement for RNA silencing by a bacterial small RNA and acceleration of duplex formation by Hfq.. Molecular Microbiology.

[10] Morita T, Maki K, Aiba H (2005). RNase E-based ribonucleoprotein complexes: mechanical basis of mRNA destabilization mediated by bacterial noncoding RNAs.. Genes Dev.

[11] Morita T, Mochizuki Y, Aiba H (2006). Translational repression is sufficient for gene silencing by bacterial small noncoding RNAs in the absence of mRNA destruction.. PNAS.

[12] Brantl S (2007). Regulatory mechanisms employed by cis-encoded antisense RNAs.. Curr Opin Microbiol.

[13] Wagner EG, Altuvia S, Romby P (2002). Antisense RNAs in bacteria and their genetic elements.. Adv Genet.

[14] Masse E, Escorcia FE, Gottesman S (2003). Coupled degradation of a small regulatory RNA and its mRNA targets in Escherichia coli.. Genes Dev.

[15] Blomberg P, Wagner EG, Nordstrom K (1990). Control of replication of plasmid R1: the duplex between the antisense RNA, CopA, and its target, CopT, is processed specifically in vivo and in vitro by RNase III.. EMBO J.

[16] Krinke L, Wulff DL (1990). The cleavage specificity of RNase III.. Nucleic Acids Res.

[17] Pertzev AV, Nicholson AW (2006). Characterization of RNA sequence determinants and antideterminants of processing reactivity for a minimal substrate of Escherichia coli ribonuclease III.. Nucl Acids Res.

[18] Udekwu KI, Darfeuille F, Vogel J, Reimegard J, Holmqvist E (2005). Hfq-dependent regulation of OmpA synthesis is mediated by an antisense RNA.. Genes Dev.

[19] Coornaert A, Lu A, Mandin P, Springer M, Gottesman S MicA sRNA links the PhoP regulon to cell envelope stress.. Molecular Microbiology.

[20] Schauder S, Shokat K, Surette MG, Bassler BL (2001). The LuxS family of bacterial autoinducers: biosynthesis of a novel quorum-sensing signal molecule.. Molecular Microbiology.

[21] Beeston AL, Surette MG (2002). pfs-Dependent Regulation of Autoinducer 2 Production in Salmonella enterica Serovar Typhimurium.. J Bacteriol.

[22] De Keersmaecker SCJ, Sonck K, Vanderleyden J (2006). Let LuxS speak up in AI-2 signaling.. Trends in Microbiology.

[23] De Lay N, Gottesman S (2009). The Crp-activated small noncoding regulatory RNA CyaR (RyeE) links nutritional status to group behavior.. J Bacteriol.

[24] Lutz R, Bujard H (1997). Independent and tight regulation of transcriptional units in Escherichia coli via the LacR/O, the TetR/O and AraC/I1-I2 regulatory elements.. Nucleic Acids Res.

[25] Udekwu KI, Wagner EGH (2007). Sigma E controls biogenesis of the antisense RNA MicA.. Nucl Acids Res.

[26] Church GM, Gilbert W (1984). Genomic Sequencing.. PNAS.

[27] Aldea M, Garrido T, Pla J, Vicente M (1990). Division genes in Escherichia coli are expressed coordinately to cell septum requirements by gearbox promoters.. EMBO J.

[28] Bensing BA, Meyer BJ, Dunny GM (1996). Sensitive detection of bacterial transcription initiation sites and differentiation from RNA processing sites in the pheromone-induced plasmid transfer system of Enterococcus faecalis.. PNAS.

[29] Bralley P, Jones GH (2004). Organization and Expression of the Polynucleotide Phosphorylase Gene (pnp) of Streptomyces: Processing of pnp Transcripts in Streptomyces antibioticus.. J Bacteriol.

[30] Papamichail D, Delihas N ( 2006) Outer membrane protein genes and their small non-coding RNA regulator genes in Photorhabdus luminescens.. Biology Direct.

[31] Winzer K, Hardie KR, Burgess N, Doherty N, Kirke DF (2002). LuxS: its role in central metabolism and the in vitro synthesis of 4-hydroxy-5-methyl-3(2H)-furanone.. Microbiology.

[32] Xavier KB, Bassler BL (2003). LuxS quorum sensing: more than just a numbers game.. Current Opinion in Microbiology.

[33] Surette MG, Miller MB, Bassler BL (1999). Quorum sensing in Escherichia coli, Salmonella typhimurium, and Vibrio harveyi: A new family of genes responsible for autoinducer production.. PNAS.

[34] Zhang M, Sun K, Sun L (2008). Regulation of autoinducer 2 production and luxS expression in a pathogenic Edwardsiella tarda strain.. Microbiology.

[35] Argaman L, Hershberg R, Vogel J, Bejerano G, Wagner EGH (2001). Novel small RNA-encoding genes in the intergenic regions of Escherichia coli.. Current Biology.

[36] Robertson HD (1982). Escherichia coli ribonuclease III cleavage sites.. Cell.

[37] Vendeville A, Winzer K, Heurlier K, Tang CM, Hardie KR (2005). MAKING ‘SENSE’ OF METABOLISM: AUTOINDUCER-2, LUXS AND PATHOGENIC BACTERIA.. Nature Reviews Microbiology.

[38] Bassler BL, Losick R (2006). Bacterially Speaking.. Cell.

[39] Wang L, Li J, March JC, Valdes JJ, Bentley WE (2005). luxS -dependent gene regulation in Escherichia coli K-12 revealed by genomic expression profiling.. J Bacteriol.

[40] Lelong C, Aguiluz K, Luche S, Kuhn L, Garin J (2007). The Crl-RpoS regulon of Escherichia coli.. Mol Cell Proteomics.

